# TrmL and TusA Are Necessary for *rpoS* and MiaA Is Required for *hfq* Expression in *Escherichia coli*

**DOI:** 10.3390/biom7020039

**Published:** 2017-05-04

**Authors:** Joseph I. Aubee, Morenike Olu, Karl M. Thompson

**Affiliations:** 1 Department of Microbiology, College of Medicine, Howard University, Washington, DC 20059, USA; joseph.aubee@howard.edu (J.I.A.); morenike.olu@bison.howard.edu (M.O.); 2 Department of Biology, College of Arts and Sciences, Howard University, Washington, DC 20059, USA

**Keywords:** s^2^U34 tRNA modification, C/U34m tRNA modification, i^6^A37 tRNA modification, RpoS, Hfq, MiaA, TrmL, TusA, leucine, codon bias

## Abstract

Previous work demonstrated that efficient RNA Polymerase sigma S-subunit (RpoS) translation requires the N6-isopentenyladenosine i6A37 transfer RNA (tRNA) modification for UUX-Leu decoding. Here we investigate the effect of two additional tRNA modification systems on RpoS translation; the analysis was also extended to another High UUX-leucine codon (HULC) protein, Host Factor for phage Qβ (Hfq). One tRNA modification, the addition of the 2’-O-methylcytidine/uridine 34 (C/U34m) tRNA modification by tRNA (cytidine/uridine-2’O)-ribose methyltransferase L (TrmL), requires the presence of the *N*^6^-isopentenyladenosine 37 (i^6^A37) and therefore it seemed possible that the defect in RpoS translation in the absence of i^6^A37 prenyl transferase (MiaA) was in fact due to the inability to add the C/U34m modification to UUX-Leu tRNAs. The second modification, addition of 2-thiouridine (s^2^U), part of (mnm^5^s^2^U34), is dependent on tRNA 2-thiouridine synthesizing protein A (TusA), previously shown to affect RpoS levels. We compared expression of P*_BAD_*-*rpoS990-lacZ* translational fusions carrying wild-type UUX leucine codons with derivatives in which UUX codons were changed to CUX codons, in the presence and absence of TrmL or TusA. The absence of these proteins, and therefore presumably the modifications they catalyze, both abolished P*_BAD_*-*rpoS990*-*lacZ* translation activity. UUX-Leu to CUX-Leu codon mutations in *rpoS* suppressed the *trmL* requirement for P*_BAD_*-*rpoS990*-*lacZ* expression. Thus, it is likely that the C/U34m and s^2^U34 tRNA modifications are necessary for full *rpoS* translation. We also measured P*_BAD_*-*hfq306-lacZ* translational fusion activity in the absence of C/U34m (*trmL*) or i^6^A37 (*miaA*). The absence of i^6^A37 resulted in decreased P*_BAD_*-*hfq306-lacZ* expression, consistent with a role for i^6^A37 tRNA modification for *hfq* translation.

## 1. Introduction

*Escherichia coli* RpoS (σ^S^) is an alternative sigma factor that facilitates physiological adaptation to general starvation and stationary phase growth [1,2]. RpoS levels are tightly regulated, particularly at the post-transcriptional level. Several small regulatory RNAs modulate translational initiation in response to various environmental conditions [3,4,5,6,7,8]. In addition, RpoS stability is decreased by the activity of the ATP-dependent Protease ClpXP with the assistance of the Regulator of sigma S B (RssB) adaptor protein [9]. Conversely, RpoS is stabilized by three anti-adaptor proteins: Inhibitor of RssB activity in response to phosphate starvation (IraP), Inhibitor of RssB activity in response to magnesium starvation (IraM), and Inhibitor of RssB activity in response to DNA damage (IraD) [10,11,12,13].

**Figure 1:**
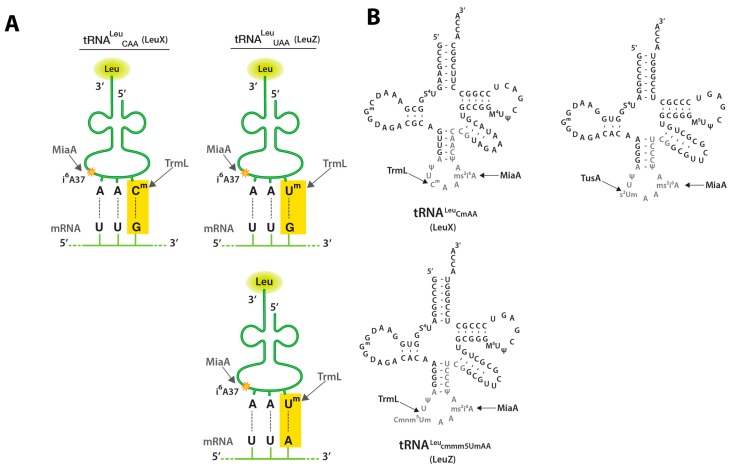
tRNA (cytidine/uridine-2’O)-ribose methyltransferase L (TrmL), tRNA dimethylallyltransferase (MiaA), and tRNA 2-thiouridine synthesizing protein A (TusA) tRNA modifications and UUX-Leu codon recognition; (**A**) Schematic depicting tRNA^Leu^_CAA_ (LeuX) and tRNA^Leu^_UAA_ (LeuZ) anticodons and their cognate mRNA codons (UUG or UUA). This schematic is modified from a leucine tRNA schematic obtained from the following source [20]; (**B**) tRNA^Leu^_CAA_ (LeuX) and tRNA^Leu^_UAA_ (LeuZ) secondary structure secondary structure. Nucleotides subject to modification are shown in grey and the sites of the MiaA, TrmL, and TusA modifications studied here are shown. This schematic is modified from Figure 3C of [21].

RpoS expression is also regulated at the level of translation within the open reading frame (ORF). SsrA (tmRNA) is a small RNA that enters the A-site of stalled ribosomes and is translated into a C-terminal tag that targets truncated proteins for degradation by several cellular proteases [14]. SsrA is necessary for full RpoS translation [14]. Translation of RpoS is significantly affected by at least one tRNA modification, *N*6-isopentyl adenosine (i^6^A) [15]. tRNA dimethylallyltransferase (MiaA) catalyzes the prenylation (i^6^) of adenine 37 (A37) to tRNAs that read UXX codons [16,17,18]. The mechanism of action of the i^6^A37 tRNA modification on RpoS expression is, at least in part, promotion of efficient UUX-leucine decoding (Figure 1) [19]. Here, we examined whether two other tRNA modification systems played roles in RpoS translation. 

A major role for tRNA modifications is the promotion of translational fidelity, through suppression of frameshifting and ribosome stalling [22,23,24]. Modifications also improve reading frame maintenance in response to rare or underutilized codons [25,26]. In this work, we investigated the role of several tRNA modifications on *rpoS* or *hfq* expression: 2′-*O*-methylation of Cytidine or Uridine, at the wobble position (C/Um), 2-thiouridine at the wobble position (s^2^U), as well as isopentyl adenosine 37 (i^6^A) (Figure 1).

C/U34m is catalyzed by the tRNA methyl transferase enzyme TrmL enzyme in *E. coli* [21,27,28]. The C/U34m modification occurs in leucine tRNA_CmAA_ and tRNA_cmnm5UmAA_ (Figure 1) [21]. Both of these leucine tRNA isoacceptors recognize UUA-Leu and UUG-Leu codons, and also carry the i^6^A37 tRNA modification that assists in the optimal decoding of the Leu codons during *rpoS* translation (Figure 1) [19]. The TrmL-catalyzed C/U34m modification of these leucine tRNAs isoacceptors requires i^6^A37 modification [21]. Taken together, this suggests that *miaA* and *trmL* may work together to affect *rpoS* expression and we investigated the role the *trmL* in *rpoS* translation in this work.

The 2-thiolation (s^2^) tRNA modification occurs in the anticodon stem loop (ASL) on uridines at the wobble position 34 (Figure 1) [29]. The 2-thiouridine (s^2^U34) wobble tRNA modification, like other tRNA modifications within the ASL, improves translational fidelity by suppressing translational frameshifting, promoting tight codon-anticodon interactions for tRNA^Gln^_UUG_ with CAA and CAG glutamine codons [30]. tRNA 2-thiouridine synthesizing protein A (TusA) is necessary for the synthesis of the s^2^U34 modification [31]. TusA point mutations were identified in a genetic screen for mutations affecting *rpoS* expression, decreasing σ^S^ levels at the level of stability [32]. In addition, the s^2^U34 modification is also a component of a more complex modification present on C/U34m modified leucine tRNA isoacceptors, one of which is implicated in *rpoS* translation [19].

Host Factor for phage Qβ (Hfq) is a co-factor necessary for the vast majority of trans-acting small regulatory RNAs in *E. coli* and other bacteria, and has many pleotropic roles in the cell [33,34]. It was first discovered as a host factor for Bacteriophage Qβ replication [35]. The *hfq* gene is in a complex operon with multiple promoters and is immediately downstream of the i^6^A37 prenyl transferase *miaA* [36]. There is an elevated UUX-Leu to CUX-Leu ratio in the *hfq* open reading frame, characterizing it as a HULC protein that may, like RpoS, be sensitive to the i^6^A37 tRNA modification [19]. There is little known about the translational regulation of the *hfq* ORF, in particular the contribution of tRNA modifications.

Here we used a genetic approach to test the role of the TrmL-catalyzed and TusA-catalyzed tRNA modifications during *rpoS* expression. We also further tested our previous predictions on the role of the i^6^A37 modification on expression of proteins with High UUX leucine Codon (HULC) content, using *hfq* as a model gene. Hfq was an attractive candidate, due to its elevated UUX-Leu codon usage ratio (Table S7, [19]) and its phylogenetically conserved cotranscription with *miaA*. Here we demonstrate that both TrmL and TusA are necessary for full RpoS translation and MiaA-catalyzed-i^6^A37 is necessary for *hfq* expression.

## 2. Results

### 2.1. Wobble Base tRNA Modifications Enzymes TrmL and TusA Are Necessary for RpoS Expression

In order to determine if mnm^5^s^2^U34 and C/U34m tRNA modifications may play a role in facilitating proper *rpoS* translation, we measured the effect of mutations in the enzymes necessary for these modifications on *rpoS* expression, using two different *rpoS*-*lacZ* translational fusion strains. Here, we will assume that phenotypes associated with absence of the enzyme are due to lack of the modification, although it is possible these enzymes have other roles in the cell.

**Figure 2:**
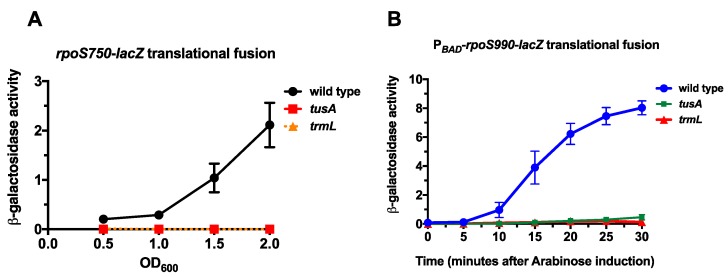
The effect of s^2^U34 and C/U34m tRNA modifications on *rpoS*-*lacZ* expression; (**A**) Wild type (EM1050), *trmL*^−^ (KMT767), and *tusA*^−^ (KMT766) *rpoS750*-*lacZ* translational fusion strains were grown in Luria Bertani (LB) Lennox media at 37 °C and 200 rpm. Aliquots were taken at Optical Density 600 nm (OD_600_) of 0.5, 1.0, 1.5, and 2.0 for β-galactosidase assay; (**B**) Wild type (KMT30003), *trmL*^−^ (KMT30003), and *tusA*^−^ (KMT30003) P*_BAD_*-*rpoS990*-*lacZ* translational fusion strains (in *rssB*^−^ backgrounds) were grown in LB media, supplemented with glucose to a final concentration of 0.2%, at 37 °C to an OD_600_ of 0.5. Cells were harvested by centrifugation and resuspended in LB media, supplemented with arabinose to a final concentration of 0.2%, and further incubated at 37 °C. Aliquots were taken at 5 min intervals for 30 min for β-galactosidase assay.

The first translational fusion strain contains the *rpoS* promoter, the 5′ untranslated region, and 750 nucleotides of the open reading frame (*rpoS750*-*lacZ*) fused, in frame, to the ninth codon of the *lacZ* ORF. The second translational fusion strain has the arabinose inducible P*_BAD_* promoter in place of the *rpoS* promoter and 5′ untranslated region (5’ UTR), as well as the complete open reading frame, except the termination codon (P*_BAD_*-*rpoS990*-*lacZ*) fused, in frame, to the ninth codon of the *lacZ* ORF. Both of these fusions were previously used to characterize the contribution of the MiaA-catalyzed i^6^A37 tRNA modification in *rpoS* translation [15,19]. We transduced zeomycin-linked null mutations in *trmL* (Δ*trmL*::*zeo*) and *tusA* (Δ*tusA*::*zeo*) into both the *rpoS750*-*lacZ* and P*_BAD_*-*rpoS990*-*lacZ* translational fusion strains and measured β-galactosidase expression compared to the wild type control strain (Figure 1B and Figure 2A). For the *rpoS750*-*lacZ* translational fusion—at Optical Density 600nm (OD_600_) 0.5, 1.0, 1.5, and 2.0—the activity of the *rpoS*-*lacZ* fusion was virtually undetectable in the absence of *trmL* or *tusA* at <1.0 machine units (Figure 2A). Arabinose led to significant induction of the wild type P*_BAD_*-*rpoS990*-*lacZ*, as expected and previously observed (Figure 2B). However, in the absence of *trmL* or *tusA* this fusion also had virtually undetectable activity, with specific activities <1.0 machine units throughout the 30 min following induction (Figure 2B). Taken together, the decreased activity of the *rpoS* fusions in the absence of *tusA* and *trmL* suggest that the presence of the s^2^U34 and C/U34m tRNA modifications are necessary for *rpoS* expression. Any effects seen with both fusions cannot be attributed to the native promoter or the 5′ UTR (and thus sRNA regulation of RpoS), since neither the native promoter nor the 5′ UTR are present in the P*_BAD_* fusion. Effects on RpoS-LacZ levels could reflect differences in translation, in mRNA stability, or in protein degradation; these possibilities are explored in the discussion.

### 2.2. TrmL tRNA Modification is Necessary for Decoding of UUX-Leucine Decoding in RpoS

**Figure 3:**
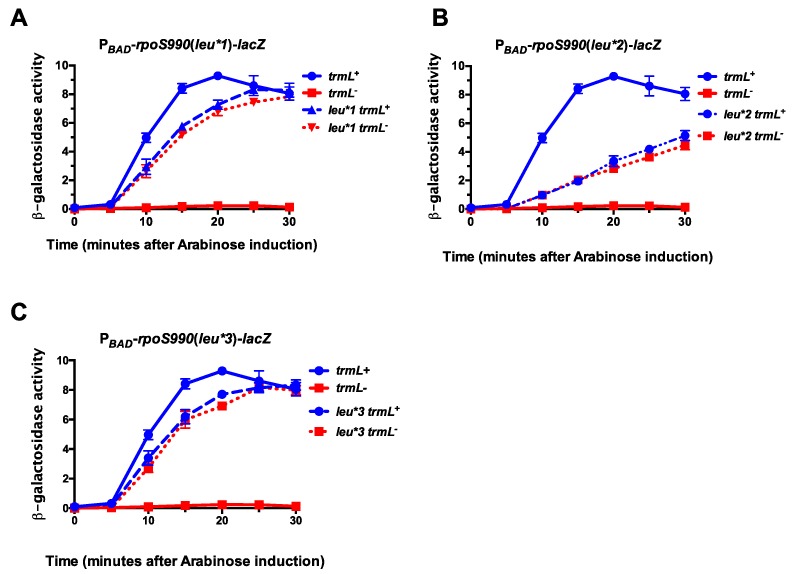
*trmL* is necessary for leucine decoding during RpoS expression. *trmL*^+^ and *trmL*^−^ P*_BAD_*-*rpoS990*-*lacZ* translational fusions (in *rssB*^−^ backgrounds) were grown in LB, supplemented with glucose to a final concentration of 0.2%, at 37 °C to an OD_600_ of 0.5. Cells were harvested by centrifugation and res-suspended in LB supplemented with arabinose to a final concentration of 0.2%. Finally, 100 mL aliquots of culture were isolated at 5-min intervals for 30 min. This process was executed for P*_BAD_*-*rpoS990*-*lacZ* translational fusions with *rpoS* UUA-Leu CUX-Leu mutations (leu*1)–strains KMT36002 and KMT36010 (**A**); *rpoS* UUG-Leu to CUX-*leu* mutation (leu*2)–strains KMT37002 and KMT37010 (**B**); *rpoS* with both UUA-Leu to CUX-Leu and UUG-Leu to CUX-Leu mutations (leu*3)–strains KMT33001 and KMT33013 (**C**). All time points represent the average of three independent experiments (biological replicates) and the error bars represent the standard error of the mean.

We previously demonstrated that the i^6^A37 tRNA modification was required for UUX-leu decoding, with silent UUX-Leu to CUX-Leu codon mutations in the *rpoS* reading frame partially suppressing the i^6^A37 requirement for expression [19]. In addition, since the C/U34m occurs in certain leucine tRNAs, requires the MiaA-catalyzed i^6^A37 tRNA modification, and may be necessary for *rpoS* translation (Figure 1), we asked whether the *trmL* requirement for RpoS expression may be related to UUX-leucine decoding, using our previously described UUX to CUX mutant derivatives of the P*_BAD_*-*rpoS990*-*lacZ* fusions [19]. We measured the activity of P*_BAD_*-*rpoS990*-*lacZ* translational fusions in which UUA-Leu codons with the *rpoS* portion of the fusion ORF have been changed to CUX-Leu (leu*1), UUG-Leu to CUX-Leu (leu*2), or UUA-Leu and UUG-Leu to CUX-Leu (leu*3) mutations, in the presence and absence of *trmL* (Figure 3). Either the UUA-Leu to CUX-Leu codon construct (leu*1) or the UUG-Leu to CUX-Leu construct (leu*2) in the *rpoS* ORF completely suppressed the *trmL* requirement for *rpoS* expression (Figure 2B and Figure 3A). Finally, combined UUA-Leu to CUX-Leu and UUG-Leu to CUX-Leu codon mutations within the *rpoS* ORF also completely suppressed the *trmL* requirement (Figure 3C). Taken together, this strongly supports the idea that the TrmL-catalyzed C/U34m tRNA modification is necessary for *rpoS* translation, dependent upon the presence of UUX-Leu codons.

### 2.3. MiaA Is Necessary, While TrmL Is Dispensable, for Hfq Expression

**Figure 4:**
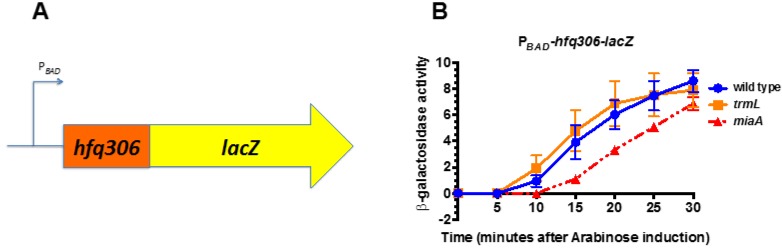
*miaA*, not *trmL*, is necessary for full *hfq* expression. (**A**) Schematic depicting the P*_BAD_*-*hfq30*6-*lacZ* translational fusion used for this experiment. (**B**) *miaA*^+^ *trmL*^+^ (KMT38000), *miaA*^+^ *trmL*-(KMT38004), and *miaA*^−^ *trmL*^+^ (KMT38002) P*_BAD_*-*hfq306*-*lacZ* translational fusion strains were grown in LB media, supplemented with glucose to a final concentration of 0.2% at 37 °C to an OD_600_ of 0.5. Cells were harvested by centrifugation and resuspended in LB media, supplemented with arabinose to a final concentration of 0.2%, and further incubated at 37 °C. Aliquots were taken at 5 min intervals for 30 min for β-galactosidase assay. All time points represent the average of three independent experiments (biological replicates) and the error bars represent the standard error of the mean.

We previously identified the UUX-Leu ratios of all open reading frames within the *E. coli* genome and proposed using this ratio as a predictor of i^6^A37 sensitivity during translation of the open reading frame [19]. We measured the effect of mutations in *miaA* on expression of another gene known to be involved in the RpoS regulatory circuitry, that also has a UUX-Leu ratio suggestive of i^6^A37 sensitivity, the RNA-chaperone Hfq. We hypothesized that additional HULC proteins may be sensitive to the presence of the C/U34m modification, since the expression of at least two predicted HULC were defective in the *miaA* mutant and the C/U34m modification requires the MiaA-catalyzed i^6^A37 modification. An arabinose inducible *hfq* fusion containing the *hfq* open reading frame, except the termination codon, was fused in-frame, with the ninth codon of the *lacZ* ORF (P_BAD_-*hfq306*-*lacZ*—Figure 4A). Next, we transduced in zeomycin-linked null mutations in *trmL* (Δ*trmL*::*zeo*) and *miaA* (Δ*miaA*::*zeo*), and measured P_BAD_-*hfq306*-*lacZ* activity in the wild-type, *trmL*, and *miaA* mutants following arabinose induction. The activity of the wild-type fusion increased in a time-dependent manner following arabinose induction (Figure 4). The *trmL* mutation had no difference in the activity of the fusion following induction. However, there were some differences in the activity of the *hfq* fusion in the absence of the absence of the MiaA-catalyzed i^6^A37 tRNA modification. Overall, expression of the fusion was slower to increase in the *miaA* mutant mutant, with significantly lower levels most obvious at 10 and 15′ (Figure 4 and Table 1).

Taken together, this suggests that while the TrmL-catalyzed C/U34m tRNA modification is dispensable for *hfq* translation, the MiaA catalyzed i^6^A37 is necessary for efficient *hfq* translation. This also provides experimental evidence that a 3^rd^ HULC protein, in addition to *rpoS* and *iraP*, requires the i^6^A37 tRNA modification, although further confirmation of a direct effect of the *miaA* mutant on *hfq* translation will require testing a version of the *hfq* fusion in which the UUX codons have been changed to CUX codons.

## 3. Discussion

### 3.1. Expanded Network of tRNA Modifications Affecting rpoS Expression and Physiological Implications

Prior to this work, there was only one report of a tRNA modification, i^6^A37, directly influencing *rpoS* translation [15,19]. Since there are multiple post-transcriptional regulators of RpoS, we hypothesized that additional tRNA modifications may be necessary for efficient translation of RpoS. Here we examined two additional tRNA modifications as possible regulators of *rpoS* expression, TusA-catalyzed s^2^U and TrmL-catalyzed C/Um.

Both of these tRNA modifications occur at the wobble position and are likely to influence *rpoS* expression through improving proper codon-anticodon interactions at the wobble position where non-canonical RNA–RNA interactions can occur. The C/Um modification occurs on leucine tRNA isoacceptor tRNA^Leu^_cmnm5s2AA_, which also contains the mnm^5^s^2^U34 tRNA modification and requires the ms^2^i^6^A37 tRNA modification [21]. The TusA catalyzed s^2^U34 modification is also a precursor for the 5-carboxymethylaminomethyl-2-thiouridine (cmnm^5^s^2^U34) tRNA hypermodification or the 5-methylaminomethyl-2-thiouridine (mnm^5^s^2^U34) tRNA modification via the Methylaminomethyl modification G/E (MnmG/E) pathway [37,38]. 

Our previous experiments suggested that the requirement for MiaA (assumed in this discussion to reflect a requirement for the i^6^A37 modification) was due to direct effects on decoding of *rpoS*. That evidence started from the observation that *rpoS*, unlike *rpoD*, was enriched for UUX leucine codons (termed here HULC for High UUX-leucine codon) [15,19]. The tRNA, tRNA^Leu^_CAA_ (encoded by *leuX*) that is the target for these modifications, acts as a multi-copy suppressor of the i^6^A37 requirement for optimal *rpoS* expression [19], consistent with UUX leucine codons limiting translation. Finally, *rpoS* codon swapping experiments, specifically changing UUX-Leu to CUX-Leu, demonstrated partial suppression of the MiaA requirement during *rpoS* expression [19], ruling out more indirect effects on translation. The presence of the TrmL-catalyzed C/Um modification on the tRNA^Leu^_CAA_ isoacceptor and the necessity of both TrmL and MiaA for complete *rpoS* translation suggest that TrmL and MiaA-catalyzed tRNA modifications could work together to optimize *rpoS* translation. This was tested here; the *trmL* requirement for *rpoS* translation in our experimental model were much more dramatic than the *miaA* requirement for *rpoS* expression for reasons that are not yet clear. However, because the *trmL* defect was fully suppressed when UUX codons in *rpoS* were changed to CUX codons (Figure 2), indirect effects of the *trmL* mutant on RpoS expression can be ruled out.

The precise physiological implications of the TrmL and MiaA effects on *rpoS* translation are still under investigation. However, previous reports offer some clues as to how these modifications have a more global impact on the cell. In long-term survival experiments, *trmL* mutants were less competitive than wild type cells, suggesting a role for *trmL* in stationary phase recovery [21]. That report is consistent with an important role for *trmL* in *rpoS* expression, since *rpoS* is necessary for stationary phase stress responses. In *E. coli*, the *miaA*_P3(HS)_ transcript is elevated under extreme heat shock, 50 °C [36]. In *Salmonella typhimurium*, *miaA* mutants lack the ability to survive at 42 °C and are sensitive to oxidative stress [39]. These reports both suggest that MiaA levels are important during heat shock. Leucine supplementation or suppression of the *leu* operon were able to suppress the sensitivity of *miaA* mutants to heat shock and oxidative stress [39]. Therefore, heat shock and/or leucine starvation may be critical conditions under which the i^6^A37, s^2^U34, and C/Um are necessary for *rpoS* translation. Heat shock could increase the translation requirement for leucine amino acids due to global protein denaturation. Under these limiting leucine conditions, proper incorporation of leucine tRNAs into the less commonly utilized UUX-Leu codons would be critical.

### 3.2. TusA Catalyzed s^2^U34 and rpoS Translation

The s^2^U requirement for *rpoS* expression, at the level of translation, adds to the current knowledge of the role that TusA plays on *rpoS* expression. Mutations in *tusA* were previously shown to decrease *rpoS* expression at the level of protein stability [32]. In this study, our *rpoS* translational fusions were in cells containing a deletion of the adaptor protein, RssB, which targets RpoS for degradation by the ClpXP protease; RpoS is stable in this strain background. Therefore, our observations suggest that *tusA* is necessary for optimal translation as well. Taken together, it would appear that TusA decreases RpoS expression at the level of stability and translation. It is also possible that defective *rpoS* translation in the absence of *trmL* leads to increased RpoS degradation. Similar to the *trmL* effect on the *rpoS* fusion, the *tusA* effect on the *rpoS* fusion was much more dramatic than the *miaA* effect for reasons that are not clear.

### 3.3. The i^6^A37 Requirement for Hfq and Implications for HULC Protein Predictive Model and Small RNA Biology

Hfq’s critical role in the action of bacterial small regulatory RNAs makes it a pleotropic effector of cellular physiology [34,40]. In this work, we examine the role of tRNA modifications in *hfq* translation at the level of the reading frame. We observed a two-fold decrease in Hfq levels in the absence of *miaA* (Figure 4), suggesting a role for i^6^A37 tRNA modification during *hfq* translation. Since Hfq levels are affected by the presence of i^6^A37, and sRNA steady state levels are affected by Hfq levels, we hypothesize that the presence of the i^6^A37 tRNA modification may be indirectly implicated in sRNA steady state levels.

Transcriptional regulation of *hfq* has been more extensively characterized than post-transcriptional regulation of *hfq* [41]. There are several *hfq* promoters within the *miaA* gene, each with different activity levels; these include promoters for vegetative growth and heat shock [36,41]. The transcripts from this superoperon appear to undergo post-transcriptional regulation in an RNaseE dependent manner [41]. Based on these transcriptional mapping studies, *hfq* should contain more than one 5′ untranslated region [36,41]. Proteins or small regulatory RNAs could target these 5′ UTRs for post-transcriptional regulation. The further examination of the *hfq* reading frame translation will also contribute additional understanding of *hfq* regulation and small RNA action. This is the first report on the role of tRNA modifications in Hfq expression.

While the identification of *hfq* as a HULC protein suggested that it may be sensitive to the presence of the MiaA, this has not previously been tested [19]. In addition, there have only been a few predicted HULC proteins tested for MiaA sensitivity [19]. Demonstrating the i^6^A37 sensitivity for an additional HULC protein, *hfq*, strengthens the predictive power of this model [19]. Interestingly, unlike *rpoS*, *trmL* mutants had no effect on *hfq* translation, suggesting that not all *miaA* effects are due to inability to add the TrmL-dependent modification (Figure 1 and Figure 3). Based on our results (Figure 4), small regulatory RNA levels and activity may be influenced by i^6^A37 tRNA modification levels. Hfq is limiting under some conditions [42]. Therefore, decreased levels of Hfq under conditions that may decrease the i^6^A37 tRNA modification, may limit small regulatory RNA levels and activity.

### 3.4. Implications for the Prokaryotic and Eukaryotic Organisms

In eukaryotic cells, tRNA modifications have been shown to play a regulatory role in cellular physiology and stress responses [25,26,43,44,45,46,47]. This includes cell cycle progression and metabolic deficiencies [25,47]. The stress responses that require tRNA modifications include global translational stress, oxidative stress, and DNA damage [25,43,44]. Since RpoS is considered a general stress adaptor for *E. coli* and other bacteria, the role of tRNA modifications in modulating its expression draws some parallels. Mycobacterial survival during hypoxia was recently shown to require tRNA modifications [48]. This suggests a broad role for tRNA modifications in allowing cells to respond to oxidative stress across the biological domains. Mitochondrial tRNA modifications are critical for the prevention of the rare genetic and neurodegenerative disorder Mitochondrial, Encephalopathy, Lactic acidosis, and Stroke (MELAS) syndrome [49]. Those with this disease lack the 5-taurinomethyl-2-thiouridine (τm^5^s^2^) on the wobble position of tRNA^LeuUUR^ [49]. Furthermore, miRNA-9/9* expression results in the post-transcriptional repression of several tRNA modification enzymes [49]. As a parallel, our observations with Hfq suggest bacterial small RNA involvement in tRNA modification biology. Interestingly, the tRNA modification in MELAS syndrome is associated with leucine decoding.

## 4. Materials and Methods

### 4.1. Strains and Oligonucleotide Primers 

All strains were derivatives of *Escherichia coli* K12 MG1655. All strains used in this study are listed in Table S1. All oligonucleotide primers are used in this study are listed in Table S2.

### 4.2. Growth Conditions and Media

All bacteria were grown in Luria Bertani (LB) Media unless otherwise stated. In experiments requiring rapid induction of arabinose inducible fusions, LB was supplemented with 0.2% Glucose (*w*/*v*) to repress the fusion, or 0.2% Arabinose (*w*/*v*) to induce it. LB agar plates supplemented with zeomycin to a final concentration of 25 μg/mL were used to select for Δ*tusA*::*zeo* and Δ*trmL*::*zeo* recombinants or transductants. M63 minimal media supplemented with glycerol, 5% sucrose, and 80 μg/mL of Xgal (M63-Gly-Suc-XG) agar plates were used for the positive selection of P*_BAD_*-*hfq306*-*lacZ*.

### 4.3. Genetic Constructions

The Δ*tusA*::*zeo* and Δ*trmL*::*zeo* mutations were created in DJ480 mini-λ::*tet* via recombineering [50] and transduced into wild type or leu* versions of *rpoS750*-*lacZ*, P*_BAD_*-*rpoS990*-*lacZ*, P*_BAD_*-*hfq306*-*lacZ* translational fusions using Bacteriophage P1. Briefly, allelic exchange substrates for Δ*tusA*::*zeo* and Δ*trmL*::*zeo* mutations were created by PCR amplification of zeomycin resistance cassette using oligonucleotide primers KT1188 and KT1189 (Table S2) as well as KT1192 and KT1193 (Table S2), containing 40 nucleotides of flanking homology to *tusA* and *trmL*, respectively. The PCR products were analyzed by agarose gel electrophoresis and purified. An overnight culture of DJ480 mini-λ::*tet* was resuspended in fresh LB and grown at 30 °C to an OD_600_ of 0.5. The culture was then shifted to 43.5 °C for 15 min. Finally, these induced cells were washed with ice-cold H_2_O and resuspended in ice-cold 10% glycerol. Approximately 100 ng of purified PCR products, corresponding to Δ*tusA*::*zeo* and Δ*trmL*::*zeo* allelic exchange substrates, were electroporated into induced DJ480 mini-λ::*tet* cells, as well as a no DNA control, using 0.1 mm cuvettes and an electroporator at setting Ec1 (Biorad). Cells were recovered in 1 mL of LB for 30–60 min and a 100 μL aliquot of cells were spread on LB agar plates supplemented with zeomycin to a final concentration of 25 μg/mL (LB-zeo). Zeomycin resistant recombinants or transductants were purified once on LB-zeo plates and twice on LB plates.

P*_BAD_*-*hfq306*-*lacZ* translational fusions were constructed using PM1800 as previously described [19]. Briefly, allelic exchange substrates corresponding to P*_BAD_*-*hfq306*-*lacZ* were PCR amplified using synthetic gBlock DNA (IDT DNA) as a template and specific oligonucleotide primers (KT1160 and KT1161). The PCR products were analyzed by agarose gel electrophoresis and purified. An overnight culture of PM1800 was resuspended in fresh LB and grown at 30 °C to an OD_600_ of 0.5. The culture was then shifted to 43.5 °C for 15 min. Finally, these induced cells were washed with ice-cold H_2_O and resuspended in ice-cold 10% glycerol. Approximately 100 ng of purified PCR products, corresponding to P*_BAD_*-*hfq306*-*lacZ* allelic exchange substrates were electroporated into induced PM1800 cells, as well as a no DNA control, using 0.1 mm cuvettes and an electroporator at setting Ec1 (Biorad). Cells were recovered in 10 mL of LB for 18 h. Recovered cultures were serially diluted down to 10^−6^ and 100 μL aliquot of serial dilutions were spread on M63-Gly-Suc-XG. Colonies that were blue on XG were purified once on M63-Gly-Suc-XG plates once and twice on LB. Then, they were screened for chloramphenicol sensitivity and confirmed by PCR.

### 4.4. β-Galactosidase Assays

High-throughput kinetic β-galactosidase assays were carried out in 96-well plates as previously described [19]. The Filtermax F5 (Molecular Devices, Sunnyvale, CA 94089 USA) multimode microplate reader was used to read microtiter plates. β-galactosidase specific activity units are defined as the slope of OD_420_ reading divided by OD_600_ and are approximately 25-fold lower than Miller Units.

Experimental design for assays executed following arabinose induction. Briefly, samples to be assayed were grown in 5 mL of LB-Glu overnight at 37 °C in a roller drum. Overnight cultures were diluted 1:1000 in 30 mL of fresh LB-Glu in a 125-mL Erlenmeyer flask and grown at 37 °C in a shaking water bath at 200 rpm. When cultures reached an OD_600_ of 1.0, cells were harvested by centrifugation, and resuspended in 30 mL of fresh LB supplemented 0.2% arabinose and 100 μL aliquots of each culture were taken every 5 min for β-galactosidase assays. Samples were collected in triplicate for each individual experiment and averages were taken as a representative sample for each experiment. The data presented represent the mean and standard error of the mean of at least three independent replicates.

## 5. Conclusions

TrmL catalyzed tRNA methylation C/U34m and TusA catalyzed tRNA thiouridinylation are critical for *rpoS* expression in *E. coli*. Removal of UUX-codons from the *rpoS* open reading frame suppresses the *rpoS* requirement for *trmL* during translation. The MiaA catalyzed i^6^A37 tRNA modification is required for full *hfq* translation in *E. coli*.

## Figures and Tables

**Table 1:** P_BAD_-*hfq306*-*lacZ* translational fusion *miaA*^+^/*miaA*^−^ activity ratio.

Time (Min after Ara Induction)	Strain (Mean β-Gal Activity)		
	*hfq*^+^	*hfq^-^*	*hfq*^+^/*hfq*^-^ Fold Change
10	0.94	0.00	∞
15	3.91	1.11	3.52
20	6.04	3.35	1.80
25	7.50	5.10	1.47
30	8.62	6.89	1.25

## Supplementary Materials

The following are available online at http://www.mdpi.com/2218-273X/7/2/39/s1, Table S1: Strain list, Table S2: Oligonucleotide list.

Supplementary File 1

Supplementary File 2
